# Performance of MRI for Detection of ≥pT1b Disease in Local Staging of Endometrial Cancer

**DOI:** 10.3390/cancers16061142

**Published:** 2024-03-13

**Authors:** Leonie Van Vynckt, Philippe Tummers, Hannelore Denys, Menekse Göker, Sigi Hendrickx, Eline Naert, Rawand Salihi, Koen Van de Vijver, Gabriëlle H. van Ramshorst, Donatienne Van Weehaeghe, Katrien Vandecasteele, Geert M. Villeirs, Pieter J. L. De Visschere

**Affiliations:** 1 Faculty of Medicine and Health Sciences, Ghent University, 9000 Ghent, Belgium; 2 Department of Obstetrics and Gynecology, Ghent University Hospital, 9000 Ghent, Belgium; 3 Department of Medical Oncology, Ghent University Hospital, 9000 Ghent, Belgium; 4 Department of Radiology and Nuclear Medicine, Ghent University Hospital, Corneel Heymanslaan 10, 9000 Ghent, Belgium; 5 Department of Pathology, Ghent University Hospital, 9000 Ghent, Belgium; 6 Department of Gastrointestinal Surgery, Ghent University Hospital, 9000 Ghent, Belgium; 7 Department of Radiation Oncology, Ghent University Hospital, 9000 Ghent, Belgium

**Keywords:** endometrial cancer, magnetic resonance imaging, MRI, staging

## Abstract

**Simple Summary:**

Magnetic resonance imaging (MRI) can be used for the preoperative local staging of endometrial cancer (EC). The purpose of this study was to assess the performance of MRI for the detection of ≥pT1b disease (i.e., tumor invasion in ≥50% of the myometrium, into the cervical stroma or spread outside the uterus) and to evaluate whether tumor size measured via MRI was predictive for ≥pT1b disease, independent of imaging signs of invasion. We found that MRI had good performance for the detection of ≥pT1b disease and that a tumor diameter of ≥40 mm and a tumor volume of ≥20 mL were highly predictive for the presence of ≥pT1b disease. Our results support the use of MRI in the preoperative staging of EC and suggest including size criteria in EC staging guidelines.

**Abstract:**

Magnetic resonance imaging (MRI) can be used for the preoperative local staging of endometrial cancer (EC). The presence of ≥pT1b disease (i.e., tumor invasion in ≥50% of the myometrium, into the cervical stroma or spread outside the uterus) has important prognostic value and implications for the decision to perform lymphadenectomy. The purpose of this study was to assess the performance of MRI for the detection of ≥pT1b disease and to evaluate whether tumor size measured via MRI was predictive for ≥pT1b disease, independent of imaging signs of deep invasion. MRI T-staging and tumor diameter and volume were correlated with histopathology of the hysterectomy specimen in 126 patients. MRI had a sensitivity, specificity, positive predictive value, negative predictive value and accuracy of 70.0%, 83.3%, 79.2%, 75.3% and 77.0%, respectively, for the detection of ≥pT1b disease. A tumor diameter of ≥40 mm and volume of ≥20 mL measured via MRI were predictive for ≥pT1b disease at rates of 78.3% and 87.1%, respectively. An EC size of at least 5 mm upon MRI was predictive for ≥pT1b disease in more than 50% of cases. Our results support the use of MRI in the preoperative staging of EC and suggest including size criteria in EC staging guidelines.

## 1. Introduction

Endometrial cancer (EC) is the most common gynecological cancer in developed countries and the sixth most common cancer among women worldwide [1,2,3]. The incidence is increasing, presumably due to the rising prevalence of risk factors such as obesity and aging of the population [2,3,4]. There are many genetic factors associated with an increased risk for EC of which the most important is Lynch Syndrome [5,6]. Most patients with EC are postmenopausal women presenting with abnormal vaginal bleeding [3,4,5,7]. The first examination performed in the diagnostic work-up is a transvaginal ultrasound (TVU). Endometrial thickness greater than 4 mm in a postmenopausal woman is suspicious and warrants histopathological sampling to confirm the diagnosis of EC [8,9]. Historically, two types of EC were defined. Type I included the most common endometroid adenocarcinomas (80–85%), further divided into three grades (well, moderately and poorly differentiated). Type II (10–15%) included non-endometrioid types such as serous, clear cell and undifferentiated tumors with a relatively worse prognosis [1,3,4,5,6,7]. Based on new developments, a molecular classification has now replaced this traditional morphological classification. The International Federation of Gynaecology and Obstetrics (FIGO) guidelines were updated in 2023 and have increased focus on these newer molecular aspects [10]. In parallel, the TNM classification (UICC 8th edition of 2017) is used for the staging of EC, in which pT1a indicates EC limited to the endometrium or <50% of the myometrium, pT1b indicates EC invasion in ≥50% of the myometrium, pT2 indicates EC invasion into the cervical stroma and pT3 or higher indicates spread outside the uterus.

The standard treatment for localized EC includes hysterectomy with bilateral salpingo-oophorectomy and bilateral sentinel lymph node (SLN) procedure for both treatment and staging purposes. When the SLN is not found or an SLN procedure is not possible, a lymphadenectomy may be performed additionally, depending on the size of the tumor or its histological type. Lymph node metastasis is the strongest predictor of recurrence and is related with aggressive histological types and ≥pT1b disease. It has been shown that ≥pT1b disease is associated with a 6-fold higher prevalence of pelvic and para-aortic lymph node metastases as compared to <50% myometrial invasion, and therefore lymph node surgery is generally recommended in these cases in addition to hysterectomy [2,7,10,11,12,13,14,15,16].

Preoperative imaging, particularly assessing the extent of myometrial invasion, cervical invasion, lymph node metastasis and distant metastasis, is used to optimize treatment decision and to tailor surgery [2,8,15,17,18]. In addition to TVU, magnetic resonance imaging (MRI), computed tomography (CT) and positron emission tomography (PET)-CT may be used for local staging and for the assessment of lymph nodes and distant metastasis [7,8,18]. MRI is the preferred imaging modality for local staging of EC [12,19]. MRI may be useful for surgical planning because when ≥pT1b disease is preoperatively visualized, the recommended lymphadenectomy can then be performed immediately with the hysterectomy, avoiding a second operation. Moreover, EC tumor size can be measured relatively easily via preoperative MRI and may be a useful biomarker in the management of EC. EC tumor size has been suggested to be an independent prognostic factor and may even predict distant failure more accurately as compared to the depth of myometrial invasion [20,21,22,23,24].

There is still some controversy about the value of routine preoperative MRI in the local staging of EC. The European Society of Urogenital Radiology (ESUR) recommends the use of MRI for the preoperative assessment of the depth of myometrial invasion [12,19], but in the current 2023 FIGO staging guidelines, MRI is not mentioned [10].

Therefore, the purpose of this study was to assess the performance of MRI for the local staging of EC, especially in the assessment of ≥pT1b disease and to evaluate whether tumor size measured via MRI was predictive for the presence of ≥pT1b disease, independent of imaging signs of invasion.

## 2. Materials and Methods

This is a retrospective analysis of all patients diagnosed with EC at our institution who underwent MRI for local staging before surgery between February 2009 and September 2022. The scans were performed according to the routine diagnostic MRI scanning protocol used at the time of the referral. For each patient, the MRI scanning protocol and imaging findings were registered. All the MRIs were reviewed by an expert urogenital radiologist with 14 years of experience, blinded for the histopathological staging. The following parameters were assessed: TNM-stage (UICC 8th edition of 2017) on MRI, invasion of the tumor into the myometrium, visualization of enlarged lymph nodes (>10 mm axis in case of a round lymph node and 8 mm short axis in case of an oval lymph node) and presence of myomas. The size of the EC was measured in latero-lateral diameter (LL), anterior–posterior diameter (AP) and cranio-caudal diameter (CC) in mm. The volume of the tumors was estimated based on three-dimensional measurements using the ellipsoid formula AP × LL × CC × 0.52. The imaging findings were correlated with the pathological TNM-stage of the hysterectomy specimen and lymphadenectomy when applicable.

For statistical analysis, IIBM SPSS Statistics version 29 was used. We grouped the staging in a binary manner with threshold ≥pT1b disease (i.e., tumor invasion in ≥50% of the myometrium, into the cervical stroma or spread outside the uterus) versus ≤pT1a disease (i.e., tumor limited to the endometrium or invasion in <50% of the myometrium). Sensitivity, specificity, positive predictive value (PPV), negative predictive value (NPV) and accuracy were calculated using a cross-table between pathological staging (≥pT1b/≤pT1a, considered as the gold standard) and MRI-based staging (≥iT1b/≤iT1a).

The largest EC diameters measured via MRI were translated into binary variables with threshold values of 5, 10, 20, 30, 40 and 50 mm. The EC volumes measured via MRI were converted into binary variables with threshold values of 3, 5, 10, 20 and 30 mL. The area under the curve (AUC) of the receiver operating characteristic (ROC) was analyzed for EC diameter and volume. The McNemar test was used to analyze the relationship between binary pathological staging (≥pT1b/≤pT1a) and binary MRI-based staging (≥iT1b/≤iT1a). The Wilcoxon signed-rank test was used to analyze the relationship between pathological staging (pT1a, pT1b, pT2, pT3a, pT3b and pT4) and MRI-based staging (iT0, iT1a, iT1b, iT2, iT3a, iT3b and iT4). Subsequently, pathological staging (≥pT1b/≤pT1a) were correlated with tumor diameters and tumor volumes by using the Wilcoxon signed-rank test. This study was approved by our institution’s ethics committee (ONZ-2022-0167).

## 3. Results

A total of 126 patients were included (Table 1). In 86.5% (109/126), the scan protocol consisted of axial, coronal and sagittal T2-weighted images (T2-WI), sagittal dynamic contrast enhanced T1-weighted images (T1-WI) and axial T1-WI. In 8.7% (11/126), triplanar T2-WI and axial non-enhanced T1-WI were scanned. In 4.8% (6/126), triplanar T2-WI and axial non-enhanced T1-WI were supplemented with axial diffusion-weighted images (DWI). Further, 66.7% (84/126) of the scans were performed on a 1.5 Tesla scanner and 33.3% (42/126) were performed on a 3.0 Tesla system.

On MRI, the EC was considered to be limited to the inner half of the myometrium in 37.3% (47/126) and suspected to invade more than half of the myometrium in 62.7% (79/126) of the patients. In 7.1% (9/126), cervical stroma invasion was suspected. In 7.2% (9/126), enlarged lymph nodes were visualized through MRI, of which 55.5% (5/9) were limited to the pelvis and 44.4% (4/9) to the pelvic and para-aortic lymph nodes. In 48.4% (61/126) of the patients, concomitant uterine myomas were present.

The mean LL diameter of the EC measured via MRI was 26.0 mm (SD 20.1 mm), the mean AP diameter was 20.4 mm (SD 17.7 mm), and the mean CC diameter was 30.4 mm (SD 23.3 mm). The mean largest diameter measured by MRI was 32.9 mm (SD 24.2 mm). The mean estimated tumor volume was 24.5 mL with a maximum of 752.1 mL. The tumor was invisible on MRI in 11.1% (14/126) (recorded as T0). According to MRI, the tumor was considered iT1a in 46.8% (59/126), iT1b in 28.6% (36/126), iT2 in 6.3% (8/126), iT3a in 4.8% (6/126), iT3b in 1.6% (2/126) and iT4 in 0.8% (1/126).

The histological type of EC was endometrioid adenocarcinoma in 90.5% (114/126) of cases, of which 56.1% (64/114) were grade 1, 28.9% (33/114) were grade 2 and 14.9% (17/114) were grade 3. Other histological types were serous carcinomas in 4.8% (6/126), carcinosarcoma in 1.6% (2/126), clear cell carcinoma in 0.8% (1/126) or a mixed type in 2.4% (3/126). The pathological T-stage was pT1a in 52.4% (66/126), pT1b in 31% (39/126), pT2 in 5.6% (7/126), pT3a in 4.0% (5/126), pT3b in 6.3% (8/126) and pT4 in 0.8% (1/126). Regarding the hysterectomy specimen, no residual tumoral tissue was found in 4% (5/126) of the patients, although the cancer was detected on prior diagnostic pipelle or curettage. They were included in the pT1a stage group.

MRI-based T-staging was identical to pathological T-staging in 57.1% of cases (72/126), but the EC stage was understaged in 28.6% (36/126) and overstaged in 14.2% (18/126) of cases (Table 2).

For the detection of ≥pT1b disease, MRI had a sensitivity of 70.0%, a specificity of 83.3%, a PPV of 79.2%, an NPV of 75.3% and an accuracy of 77.0%.

An EC size (largest diameter measured) on MRI of >5 mm had a positive predictive value of ≥pT1b disease in 51.8%, irrespective of macroscopic visual EC extent in the outer half of the myometrium. Similarly, a maximum tumor diameter measured via MRI of >10, >20, >30, >40 and >50 mm had a positive predictive value of ≥pT1b disease in 52.3%, 58.0%, 60.3%, 78.3% and 88.0%, respectively (Table 3). Of the 14 invisible tumors on MRI, 11 (78.6%) were histologically pT1a. There were two tumors with a diameter between 0 and 5 mm on MRI, and they were both pT1a. 

An EC volume of >3 mL had a positive predictive value of 61.0% for ≥pT1b disease, irrespective of macroscopically visible EC extent in the outer half of the myometrium. Similarly, an EC volume of >5, >10, >20 and >30 mL had a positive predictive value for ≥pT1b disease of 62.5%, 72.3%, 87.1% and 91.7%, respectively (Table 4).

**Figure 1:**
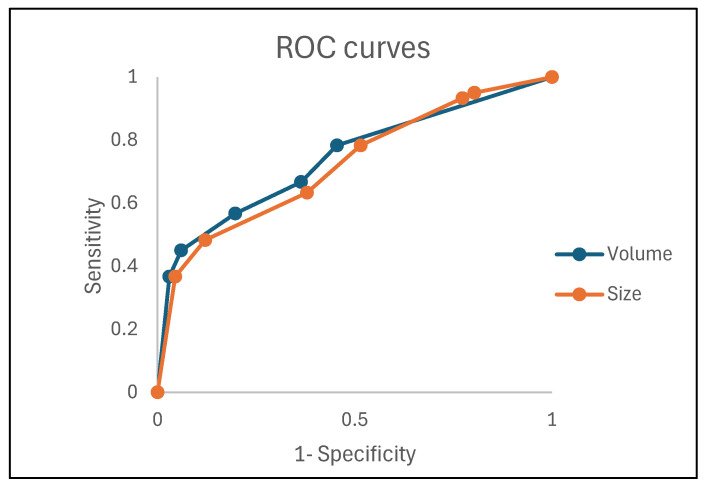
Receiver operator characteristic (ROC) curves for tumor size as determined by the greatest diameter (orange curve) and tumor volume (blue curve) in the assessment of ≥pT1b disease. The greatest area under the curve (AUC) was found with a threshold of 40 mm for tumor size (AUC 0.77) and a threshold of 20 mL for tumor volume (AUC 0.70).

The AUC of the ROC analysis for tumor size was highest at a threshold of 40 mm (0.77) ) and 20 mL (0.70) (Figure 1).

When complete pathological staging (pT1a, pT1b, pT2, pT3a, pT3b, Tp4) was correlated with complete MRI-based staging (0, pT1a, pT1b, pT2, pT3a, pT3b, Tp4), a significant relationship was observed (*p* < 0.05). However, when the correlation was examined between binary pathological staging (≥pT1b/≤pT1a) and binary MRI staging (≥pT1b/≤pT1a), this correlation did not reach statistical significance (*p* > 0.05).

The tumor diameters and volumes were significantly larger for patients with a disease stage of 1b or higher as compared to ≤pT1a (Table 5).

## 4. Discussion

In this study, we evaluated the performance of MRI for the local staging of EC. We showed that MRI had good performance for the detection of ≥pT1b disease and that a larger tumor diameter and volume were predictive for ≥pT1b disease, irrespective of imaging signs of tumor invasion into the outer half of the myometrium, the cervical stroma or outside the uterus upon MRI. We grouped the T-staging in a binary manner with threshold ≥pT1b disease, which included all ECs with ≥50% myometrial invasion (i.e., the real pT1b tumors) but also all stage pT2 or higher tumors (i.e., cervical stromal invasion or spread outside the uterus) that are not necessarily associated with EC invasion in the outer half of the myometrium. This was reasonable because the ≥pT1b threshold is the most relevant clinical factor in T-staging. It has important prognostic value and direct implications for the decision to perform lymphadenectomy in addition to hysterectomy. After diagnosing EC through a biopsy (pipelle or curettage), the most determining factors for management are aggressive histological type, ≥pT1b disease and the presence of metastatic lymph nodes. They are associated with a poorer prognosis, and therefore lymphadenectomy is recommended in addition to hysterectomy in these cases [2,7,10,12,13,14,15,16,21,25]. Pelvic and para-aortic lymph node resection holds a risk of lymphocele and/or lower limb oedema [23]. The sentinel lymph node (SLN) has now become the new standard procedure as it may eliminate the need for extensive lymphadenectomy without risks of suboptimal treatment. When the SLN is not found, the decision to perform lymphadenectomy is based on the histological aggressiveness of the tumor or the presence of ≥pT1b disease [2,7,8,10,12,13,14,15,16,21,25]. We found in our study that an EC size of at least 5 mm measured via preoperative MRI was associated with ≥pT1b disease in more than 50% of cases, which supports the strategy of performing an SLN procedure in all patients with EC.

**Figure 2:**
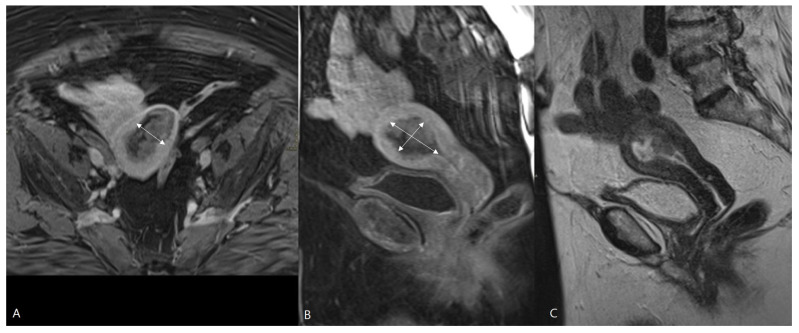
MRI of a pT1b endometrial cancer. T1-weighted images after Gadolinium administration in the axial (**A**) and sagittal plane (**B**). Sagittal T2-weighted image (**C**). The tumor is demonstrated as a heterogenous mass in the uterine cavity, with heterogenous contrast enhancement lower than the surrounding myometrium. The tumor is macroscopically invading the outer half of the myometrium, staged iT1b and confirmed histopathologically pT1b at hysterectomy. The size of the tumor was measured in three directions (white arrows) via MRI. The largest tumor diameter was assessed, and the tumor volume was calculated by the ellipsoid formula (AP × CC × LL × 0.52).

MRI has long been established as a valuable imaging method in the preoperative staging of EC. MRI can assess the depth of myometrial invasion, but histological type and grade can only be determined with endometrial tumor sampling (Figure 2). Preoperative staging with MRI may be advantageous to predict ≥pT1b disease preoperatively to avoid a second surgical procedure for lymphadenectomy [12], but there is ongoing discussion regarding the value of routine MRI in the preoperative assessment of EC [18,26]. 

The British Gynaecological Cancer Society recommends pelvic MRI in patients with histologically high-risk EC but considers MRI as optional in patients with lower-risk tumors [9]. They state that MRI should be performed according to dedicated scan protocols and should ideally be interpreted by radiologists with expertise in gynecological oncological imaging. The ESGO-ESTRO-ESP (European Society of Gynaecological Oncology–European Society for Radiotherapy and Oncology–European Society of Pathology) guidelines mention MRI as alternative for TVU but report that both imaging techniques have similar performance for detecting myometrial invasion [27]. The European Society of Urogenital Radiology (ESUR) recommends performing MRI for the local staging of EC and published guidelines describing the indications and prerequisites for a high-quality MRI exam protocol and radiological report [12,19]. In the recent 2023 update of the FIGO staging guidelines, MRI is not mentioned [10]. In the clinical practice guidelines of the Society of Gynaecologic Oncology [28], MRI is not recommended for local invasiveness but is only mentioned as an alternative imaging technique to CT or PET-CT for the identification of metastatic lymph nodes or distant metastasis. In clinical practice, the use of MRI for staging EC appears to be limited, according to two recently published papers based on online surveys completed by expert radiologists and radiology residents [29,30]. In the meta-analyses of Bi et al. [16] and Alcazar et al. [26], both evaluating the diagnostic accuracy of MRI in local staging of EC, only 14 and 8 studies, respectively, could be included, again indicating a rather low use of MRI for the staging of EC worldwide.

Reasons for the ongoing discussion and limited use of MRI in clinical practice may be the costs and limited availability of MRI in many institutions. It is also argued that local staging is sufficient with TVU and that MRI may have no added value. TVU has the advantage that it is cheaper than MRI and is readily available for the gynecologist, but the quality is dependent on the skills of the examiner [27]. The reported sensitivities, specificities and accuracies for detection of deep myometrial invasion with TVU are 71–85%, 72–90% and 72–84%, respectively [18]. The reported sensitivities, specificities and accuracies of MRI for the detection of deep myometrial invasion vary largely in the literature between 33 and 100%, 44 and 100% and 58 and100%, respectively [15,18]. In the meta-analysis of Bi et al., a pooled sensitivity of 83% and pooled specificity of 82% were reported [16]. In the meta-analysis of Alcazar et al., a sensitivity of 79% and a specificity of 81% were reported [26]. Hashimoto et al. [15] reported a sensitivity of MRI for deep myometrial invasion of 65.1%. These numbers are in line with the results of our study where we found a sensitivity of 70.0%, a specificity of 83.3% and an accuracy of 77.0% for the detection of ≥pT1b disease with MRI.

In the meta-analysis of Alcazar et al. [26], the sensitivity for detecting deep myometrial invasion was higher with MRI as compared to TVU (83% vs. 75%), but it was not statistically significant, and the specificity was similar (82%) for both techniques.

**Figure 3:**
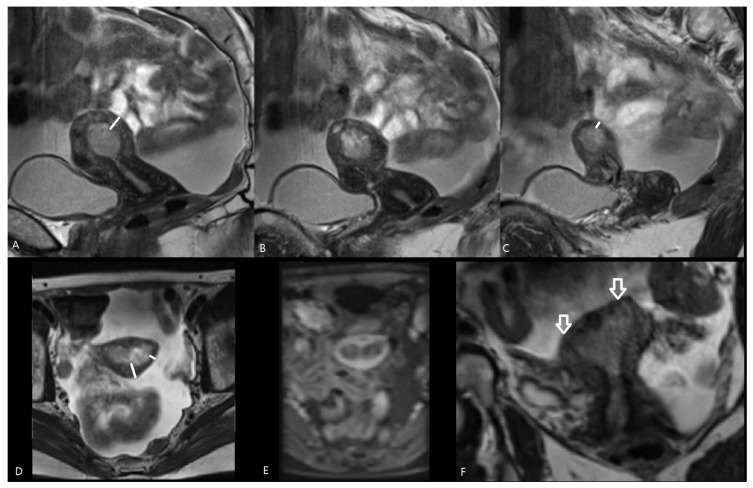
MRI of endometrial cancer. Sagittal T2-WI (**A**–**C**), axial T2-WI (**D**), axial T1-WI after Gadolinium administration (**E**) and coronal T2-WI (**F**). The tumor is extending intraluminal into the left and right cornu (white arrows on (**F**)). In the cornua, the myometrium is anatomically thinner (short white line in (**C**,**D**)) than in the uterine corpus (long white line in (**A**,**D**)), which may result in overstaging because intracavitary growth in the cornu (pT1a) may mimic tumor invasion in the outer half of the myometrium (iT1b).

**Figure 4:**
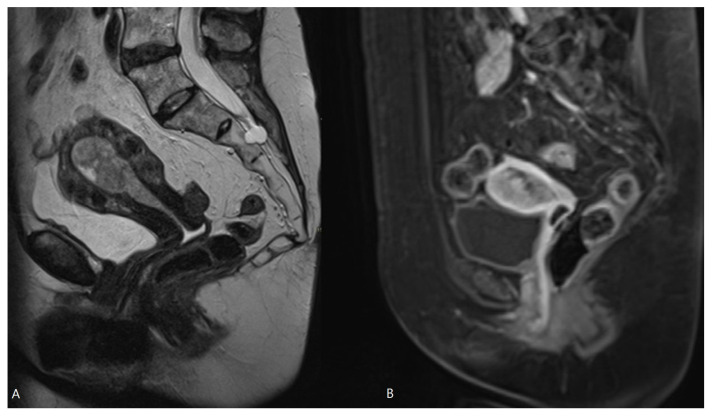
MRI of EC with bulging in the endocervical canal. Sagittal T2-WI (**A**) and sagittal T1-WI after Gadolinium administration (**B**). Histopathologically, this was pT1b disease, but in MRI there is risk of overstaging because tumor extension into the endocervical canal does not account for stage T2, which is preserved for cervical stromal invasion.

Hashimoto et al. [15] reported that preoperative MRI-based cancer stages and postoperative histopathological cancer stages were concordant in 70.0% of patients. The EC stage was underdiagnosed in MRI in 21.7% and overstaged in 8.2% of patients. This larger risk of understaging than overstaging with MRI was also observed in our study: the MRI-based T-stage was identical to the pathological T-stage in 57.1%, but the EC stage was underdiagnosed via MRI in 28.6% of patients and overstaged in 14.2% of patients. There are several causes reducing the accuracy of MRI in the local staging of EC. First, estimation of the depth of myometrial invasion is often difficult using MRI as the uterus grows atrophic in postmenopausal patients with an ill-defined junctional zone, hampering measurement of myometrial depth invasion. Moreover, in case of large or polypoid EC, or in the presence of concomitant leiomyomas or adenomyosis, myometrial compression by mass effect may further reduce the tumor-to-myometrium contrast, hampering image interpretation [8,11]. A polypoid EC bulging into the endocervical canal may be mistaken for a cervical stromal invasion, or an EC located intracavitary in the cornu may resemble tumor invasion in the outer half of the myometrium (Figure 3 and Figure 4).

The secondary aim of our study was to investigate the correlation between the size and volume of EC measured via MRI and pathological staging. Our results showed that the PPV for ≥pT1b disease increased with increasing tumor diameter and volume, independent of imaging signs of deep myometrial invasion, cervical stromal invasion or spread outside the uterus. An EC diameter of 40 mm was predictive of ≥pT1b disease in 78.3% of cases, and an EC volume of >20 mL was predictive of ≥pT1b disease in 87% of cases. It was also observed that ≥pT1b disease was present in 51.8% of the cases in which EC size in MRI was only 5 mm. Our data support the results that have been reported in the literature indicating that tumor size is relevant and may be an independent prognostic factor of EC. Chattopadhyay et al. [21] showed that in stage I tumors, size is an independent and even a better predictor than myometrial invasion for distant failure and death of EC. A size cutoff of 3.75 cm was a prognostic indicator for distant failure with a sensitivity of 67%, specificity of 72% and high negative predictive value of 98%. Canlorbe et al. [22] reported that a threshold of 35 mm in diameter had the strongest correlation with nodal metastases and relapse-free survival in women with low-risk EC but not in women with intermediate/high-risk EC. Tumor size in these studies was, however, measured using the pathology specimen and not using MRI. Ytre-Hauge et al. [20] showed that tumor volume, based on the three-dimensional measurements from the preoperative MRI, equally had predictive value for myometrial and lymph node invasion and was a prognostic factor for EC. They reported that an anteroposterior tumor diameter >2 cm significantly predicted deep myometrial invasion and that a craniocaudal tumor diameter >4 cm significantly predicted lymph node metastases. Both size parameters were significantly associated with recurrence- and progression-free survival. Lopez-Gonzalez [24] analyzed tumor volume using MRI in 127 patients with type I EC and showed that tumor volume was significantly higher for deep myometrial invasion, cervical stromal involvement, infiltrated serosa, lymph node metastases, high-grade EC, lymphovascular invasion, advanced FIGO stage and recurrence (*p* < 0.001). A tumor volume of 10 cm^3^ predicted deep myometrial invasion with a sensitivity of 82% and a specificity of 74%. ROC analysis showed that a tumor volume >25 cm^3^ predicted lymph node metastases. A volume >17 cm^3^ was associated with reduced disease-free survival and overall survival. Coronado et al. [23] reported that EC tumor volume measured via MRI was associated with aggressive histological types, deep myometrial invasion, metastatic lymph nodes and advanced FIGO stage. Five-year disease-free survival and overall survival were significantly lower at a tumor volume cut-off of ≥10 cm^3^ (69.3% vs. 84.5% and 75.4% vs. 96.1%, respectively).

The prognostic impact of tumor size in EC is thus consistently reported with cut-off values of unfavorable tumor size and volume measured via preoperative MRI in the range of our own results, around 35–40 mm and 20–25 mL, respectively. Despite this evidence, it is remarkable that the FIGO guidelines and TNM staging systems do not take the size of EC into account for staging, management or prognosis. Tumor size is, however, a factor that determines the stage and is taken into account for management in many other types of cancer, such as breast, uterine cervix and lung, to name a few [31]. The most recent FIGO guidelines of 2023 [10] are increasingly focused on molecular biology and emphasize the pathological histological classification of tumors, but staging based on tumor size or tumor volume is neither recommended nor discussed. They retain the importance of the percentage of depth of myometrial invasion, although it can be questioned why EC would suddenly behave different once it invades more than half of the myometrium [10]. Tumor size is a relatively easy and reproducible parameter to measure, and it is easier than determining the 50% threshold of myometrial invasion via MRI as well as via histopathology. A single size diameter cannot always accurately describe the exact size of a tumor; therefore, like other researchers [23,24], we estimated tumor volume in our study by applying the ellipsoid formula based on three perpendicular maximum diameters. There are currently many technological advancements available to allow for more fast, easy and accurate automatic calculations of tumor volumes in 3D. In our opinion, it should be considered to implement the long-standing suggestions regarding the use of tumor dimensions in future updates of the FIGO EC guidelines. Based on our study results and in accordance with the above-mentioned evidence in the literature [20,21,22,23,24], it could be recommended to include a tumor diameter larger than 40 mm or tumor volume larger than 20 mL as additional criteria in the selection of patients for pelvic lymph node dissection. Tumor size may be measured via TVU or MRI, but MRI may have added value in the case of small tumors with doubtful findings on TVU or for more accurately measuring tumor size and detection of imaging signs of ≥pT1b disease. In the case of larger tumors with extensive invasion outside the uterus, MRI may have added value over TVU for surgical planning and lymph node detection. Currently, there is rapidly increasing interest in the use of artificial intelligence that may have the potential to improve the imaging of endometrial cancer, e.g., to automatically delineate and measure EC or to discriminate benign conditions from EC. Radiomic analysis has been reported to have value in MRI and is currently being investigated in transvaginal ultrasound [32,33].

Our study has some limitations. First, this is a retrospective study with different scanning protocols and magnetic field strengths as used over the years. The most frequently used protocol in our study was T2-WI in three planes combined with sagittal DCE. According to the ESUR guidelines [12], the recommended scanning protocol for staging local disease in EC includes T2, DCE and DWI, but equal accuracies have been reported with protocols omitting DCE or DWI [2,7,8,11,12,13,16,17,26,34]. It has been advocated that DCE may be safely omitted when including DWI in patients in whom Gadolinium is contraindicated [18], which was the case in 4.8% (6/126) of the patients in our study. Secondly, this was a single-center study, and patients were only included if MRI was performed at our institution, but this had the advantage that all the images were readily available and could be reassessed by a local expert reader. We made an effort to have all the MRI scans reviewed by this expert urogenital radiologist in order to harmonize the radiological interpretation and size measurement, instead of just relying on the findings as described in the initial radiological report. Nevertheless, another reader may have mild variation in image interpretation and measuring the tumor diameters; therefore, a suggestion for future research is to include more readers, preferably experts but equally less-experienced readers to quantify interobserver variability.

A final limitation of our study is that we had no prognostic features such as time to recurrence or survival rates available to directly correlate the MRI imaging interpretation and size measurements with prognostic factors. One of the reasons is that the patients were scanned between 2009 and 2022 and that long-term follow up is lacking. Future research should evaluate the direct prognostic value of tumor size and volume, preferably in a multivariate analysis.

## 5. Conclusions

MRI had good performance for the detection of ≥pT1b disease in the local staging of EC.

A tumor diameter of ≥40 mm and volume of ≥20 mL measured via MRI were predictive for ≥pT1b disease in 78.3% and 87.1% of patients, respectively. An EC size of at least 5 mm upon MRI was associated with ≥pT1b disease in more than 50% of cases. Our results support the use of MRI in the preoperative staging of patients with EC and suggest including size criteria in EC staging guidelines.

## Figures and Tables

**Table 1:** Patients included in the study.

Age at Diagnosis			Mean 67 Year [SD 10]
Histological type	Endometrioid carcinoma		90.5% (N = 114)
	Grade 1		56.1% (N = 64)
	Grade 2		28.9% (N = 33)
	Grade 3		14.9% (N = 17)
	Clear cell carcinoma		0.8% (N = 1)
	Serous cell carcinoma		4.8% (N = 6)
	Carcinosarcoma		1.6% (N = 2)
	Mixed		2.4% (N = 3)
Pathological T-stage	pT1a		52.3% (N = 66)
	pT1b		31.0% (N = 39)
	pT2		5.6% (N = 7)
	pT3a		4.0% (N = 5)
	pT3b		6.3% (N = 8)
	pT4		0.8% (N = 1)
Pathological N-stage	pN0		9.4% (N = 119)
	pN1		5.6% (N = 7)
	pN2		0.0% (N = 0)
MRI technical features	Scan protocol	Axial T2-WI, coronal T2-WI, sagittal T2-WI, axial T1-WI	8.7% (N = 11)
		Axial T2-WI, coronal T2-WI, sagittal T2-WI, axial T1-WI supplemented with dynamic contrast-enhanced imaging	86.5% (N = 109)
		Axial T2-WI, coronal T2-WI, sagittal T2-WI, axial T1-WI supplemented with diffusion-weighted images (DWI)	4.8% (N = 6)
	Scanner magnetic field strength	1.5 Tesla	66.7% (N = 84)
		3.0 Tesla	33.3% (N = 42)
MRI findings	Tumor not visible		11.0% (N = 14)
	Largest diameter tumor		Mean 32.9 mm [SD 24.2]
	Volume tumor		Mean 24.5 mL [SD 74.3]
	Invasion junctional zone myometrium		67.5% (N = 85)
	Deep myometrial invasion (≥50%)		62.7% (N = 79)
	No signs of cervical stromal invasion		91.3% (N = 115)
	Doubtful cervical stromal invasion		1.6% (N = 2)
	Obvious cervical stromal invasion		7.1% (N = 9)
	Myomas		48.4% (N = 61)
	No enlarged lymph nodes		92.8% (N = 117)
	Enlarged pelvic lymph nodes		4.0% (N = 5)
	Enlarged pelvic and para-aortic lymph nodes		3.2% (N = 4)
Total			126

**Table 2:** Pathological and MRI-based T-staging.

		pT Histopathology (n, %)						
		1a	1b	2	3a	3b	4	Total
iT MRI (n, %)	0 *	11 (8.7%)	1 (0.8%)	0	1 (0.8%)	1 (0.8%)	0	14 (11.1%)
	1a	44 (34.9%)	9 (7.1%)	3 (2.4%)	1 (0.8%)	2 (1.6%)	0	59 (46.8%)
	1b	9 (7.1%)	23 (18.3%)	1 (0.8%)	1 (0.8%)	2 (1.6%)	0	36 (28.6%)
	2	1 (0.8%)	3 (2.4%)	2 (1.6%)	0	2 (1.6%)	0	8 (6.3%)
	3a	1 (0.8%)	2 (1.6%)	0	2 (1.6%)	1 (0.8%)	0	6 (4.8%)
	3b	0	1 (0.8%)	1 (0.8%)	0	0	0	2 (1.6%)
	4	0	0	0	0	0	1 (0.8%)	1 (0.8%)
	Total	66 (52.4%)	39 (31.0%)	7 (5.6%)	5 (4.0%)	8 (6.3%)	1 (0.8%)	126

In green the identical iT MRI and pT Histopathology staging is highlighted, * No tumor visible on MRI.

**Table 3:** Threshold EC tumor size (largest diameter measured on MRI) and presence of ≥pT1b disease.

		Sensitivity (%)	Specificity (%)	PPV (%)	NPV (%)	AUC (ROC-Analysis)
Threshold diameter	>5 mm	95.0	19.7	51.8	81.3	0.57
	>10 mm	93.3	22.7	52.3	78.9	0.57
	>20 mm	78.3	48.5	58.0	71.1	0.61
	>30 mm	63.3	62.1	60.3	65.1	0.63
	>40 mm	48.3	87.9	78.3	65.7	0.68
	>50 mm	36.7	95.5	88.0	62.8	0.66

In green the highest AUC is highlighted.

**Table 4:** Threshold EC tumor volume (calculated via MRI using the ellipsoid formula based on measurements in three directions) and presence of ≥pT1b disease.

		Sensitivity (%)	Specificity (%)	PPV (%)	NPV (%)	AUC (ROC-Analysis)
Threshold volume	>3 mL	78.3	54.5	61.0	73.5	0.66
	>5 mL	66.7	63.6	62.5	67.7	0.65
	>10 mL	56.7	80.3	72.3	67.1	0.69
	>20 mL	45.0	94.0	87.1	65.2	0.70
	>30 mL	36.7	97.0	91.7	62.7	0.67

In green the highest AUC is highlighted.

**Table 5:** Correlation of pathological T stage with Tumor Size and Volume.

Pathological T-Stage	N	Tumor Size (Largest Diameter)	Tumor Volume
≤pT1	66	23.2 mm (SD 17.1 mm)	7.4 mL (SD 17.3 mL)
≥pT1b	60	43.5 mm (SD 25.5 mm)	43.3 mL (SD 103.5 mL)
		*p*-value < 0.001	*p*-value < 0.001

## Data Availability

The data presented in this study are available on request from the corresponding author. The data are not publicly available due to privacy and ethical restrictions.
